# Foreign Summary

**Published:** 1839-03-16

**Authors:** 


### FOREIGN SUMMARY.

*Case of Abdominal Abscess opening externally, and communicating also with the Stomach. With Remarks.* By Professor Graves.—Catharine Delany, aged fifty-six, a washerwoman, was admitted into the Meath Hospital, on the 5th of May, 1833; she had a very large abdominal tumour, which made its appearance about two years previously, and was first perceived in the left hypochondriac region. It slowly but gradually increased in size, and did not appear to affect her health, for she was able to work until a few days before her admission. The tumour was globular, felt uneven and rather solid, and was well defined in its outline; occupying the whole of the umbilical, extending upwards into the inferior portion of the epigastric, and downwards into the superior portion of the pubic region. Laterally it stretched considerably into the right and left lumbar regions. It was quite moveable, and always fell towards the side on which she lay. It had lately, *and but lately*, become painful and tender, particularly about the navel.

The length of time this tumour had been growing, its shape, and the absence of all constitutional affection, or local pain, during so long a period, induced me to consider it as ovarian. Shortly after her admission, matters began to wear a more threatening aspect; the tenderness and pain felt in the tumour increased daily, and she now was troubled with frequent returns of nausea, which in the course of a fortnight was succeeded by obstinate vomiting.

The tumour began to grow red and softer in the umbilical region, where a deep-seated fluctuation was recognisable, which soon became quite evident and superficial, accompanied by heat and deep redness of the integuments, and a surrounding hard margin. In fact, every thing announced a collection of matter rapidly making its way to the surface. In consultation it was determined not to open this, for several reasons, the principal of which was, the long continuance of the local disease seemed to preclude all hopes of ultimate recovery: in the meantime the pain, emaciation, and suffering of the poor patient increased, and while the central softening of the tumour rapidly progressed, its circumscribed and solid structure towards the circumference as rapidly subsided, so that although the bulk of the whole was probably the same, its shape and prominent appearance were much altered. The vomiting became more distressing, nothing was retained in the stomach, large quantities of fluid deeply tinged with bile were thrown up for a week or ten days; about the 8th or 9th of June, the fluid ejected suddenly changed its character, being now a thick, viscid, and glairy mucus. On the 13th the tumour burst, and continued to discharge daily nearly a gallon of fluid exactly similar to what she had lately vomited. The external opening evidently communicated with the stomach, for the moment any fluid was swallowed a portion of it was forced out through the former. On one occasion a piece of orange, which she had chewed and swallowed, blocked up the external orifice for several hours. It is well worthy of notice, that notwithstanding the deplorable ravages committed on her organs of digestion, and notwithstanding the existence of a perforation in her stomach, the tongue continued, throughout the whole of her illness, clean and moist! Again, when the perforation had taken place, the vomiting ceased; and although her most urgent sensation was that of thirst, yet she had a tolerably good appetite, which she sought continually to gratify by swallowing jelly, &c.! She lived four days after the tumour burst externally, and about nine days after the occurrence of the perforation of the stomach. The external orifice communicated with a very large sac, the seat of the abscess, and formerly, in all probability, the sac of the tumour before it had begun to suppurate. This sac extended over the whole space formerly described as occupied by the tumour, and contained a considerable quantity of thick gruel-like fluid. No solid matter whatsoever was found within the limits of the tumour; nothing remained but the sac, thickened by inflammation, and adhering by pseudo-membranes to all the neighbouring viscera. The intestines and great omentum, matted together, formed the posterior wall of the sac, but on account of the diseased state of the parts it was impossible to determine with certainty, whether the anterior wall was formed by the peritoneum lining the abdominal muscles, or by the sheath of the recti. The former supposition seems the most probable, for a large portion of the surface of the liver was within the cavity of the abscess, and at its inferior edge, was destroyed by ulceration. The opening into the stomach was in its greater curvature, and was distant from the pylorus about an inch and a half, and with the loss of substance in the liver was the result of simple ulceration, without preceding scirrhus. All the intestines and viscera behind the tumour were, without exception, free from disease. I cannot conjecture in what structure this disease originated, or what was its nature at the commencement, but it may be doubted whether an operation for letting out the matter might not have prolonged, if not saved the patient's life, had it been undertaken at the time fluctuation first became perceptible, and before the ulceration of the stomach and liver had commenced. The details I have given may possibly serve as a guide to others, should another such case occur.—*Dublin Journal, for January.*

---

*On Inflammation of the Spinal Marrow.* By Prof. Graves.—This disease is closely connected with the subject of neuralgia. *Myelitis* is so liable to be confounded with a great variety of painful affections, that every ascertained case of inflammation of the spinal marrow ought to be recorded for the purpose of rendering more perfect a department of pathology already diligently, but not completely cultivated. A young married woman, named ———, was admitted into the Meath Hospital, on the 12th September, 1838. She was healthy until the period of marriage, soon after which her husband commenced a system of ill-usage, comprising beating, kicking, throwing down stairs, &c. &c He was frequently drunk, and occasioned her every species of grief. No wonder that a life like this should have reduced our patient to the truly miserable condition she was in. She had been injured so often that it was difficult to say to what particular act of violence her present malady ought to be referred. She is much emaciated, respirations hurried, and pulse very quick. Has no headache, but complains much of agonizing pains in the loins, aggravated by pressure of the lumbar spinous processes, extending round the abdomen, and downwards to the hips and thighs. There is no pectoral affection, and her tongue, state of stomach, and general appearance, are not those of a person labouring under fever. She writhes in the bed from the violence of the pains; she does not sleep night or day, and disturbs the other patients by her cries.

Blood was drawn by cupping from the loins; leeches were applied, and Dover's powder administered. Her extreme emaciation prevented us from adopting either more active depletion by the lancet, or the use of calomel. In short, we sought to relieve, not to cure, for her death appeared inevitable. Blisters we could not apply, on account of the great emaciation. On the 15th we found that she had been screeching all night, and constantly wanting extract of opium, which was ordered her as a palliative. On the 16th she complained that the sense of feeling was leaving her thighs, and she died on the 18th, five days after admission. *On dissection* we found all the viscera healthy; there was extreme atrophy of the intestines, especially the colon and cæcum, so it is probable that starvation was among her afflictions. The lower portion of the spinal marrow, and the *cauda equina*, exhibited an excessive vascularity and redness, but no exudation of lymph. Each nervous fasciculus of the cauda exhibited a vein on its posterior surface distended with blood; and the remaining portion of each fasciculus displayed great arterial vascularity. For this case I am indebted to Mr. Brady, a practising pupil of great promise.—*Ibid.*

---

*On Neuralgia of the Testicle.* By Professor Graves.—This is not a very common form of disease, but it requires notice, as it gives rise to excruciating agony, and constitutes one of the most painful affections that can be imagined. I have seen two examples of it within the last year; the first was a young gentleman of highly irritable nerves, who had studied hard and dissipated much; in him the paroxysms of pain did not observe any very marked period, but returned daily at uncertain intervals, which grew shorter and shorter, until at last he had scarcely any respite day or night. There was no fever, and not the slightest appearance of local congestion or inflammation. When attacked with a paroxysm the patient would throw himself on the floor, and roll about in the greatest agony, covered with a cold perspiration. This case yielded to large doses of carbonate of iron freshly prepared, and frequent inunction of the testicle and cord with belladonna ointment. The second case of neuralgia of the testicle occurred in a gentleman who laboured under neuralgic pains, decidedly of a gouty nature. In him the pain of the cord and testicles used to come on every afternoon about four o'clock, and continued for several hours. The pain, though considerable, did not approach the degree of agony experienced in the first case. It was at times, however, so severe as to compel him to groan aloud. This neuralgia of the testicle disappeared after a few days, and was replaced by a violent gouty pain in the loins and right hypochondrium. The latter yielded to the usual local treatment, and the use of colchicum internally.—*Ibid.*

---

*Of a peculiarity of structure occasionally occurring in the Basilar Artery of Man.* By John Davy, M. D., F. R. S., Assistant Inspector of Army Hospitals.—Besides those peculiarities of structure of the basilar artery which are well known, there is one of not uncommon occurrence, which, to the best of my knowledge, has not hitherto been noticed by any anatomist. It is a band in the interior of the vessel, attached to its side, and consequently intersecting it. It varies both in its dimensions and situation. I have most frequently found it near the junction of the vertebral arteries; very seldom near the commencement of the circle of Willis. Sometimes the band perfectly, at other times only partially, intersects the vessel. Sometimes I have seen it not more than one line thick, occasionally two or three lines. Its appearance, as regards its nature, has always been similar and most analogous to a fibrous structure. In every instance, I apprehend, it may be considered as congenital, and not the effect of disease. This is inferred after careful examination to endeavour to detect the effects of diseased action. In no instance were any indications of such action observed. The contiguous lining membrane was smooth, and there was no thickening where the ends of the band were inserted.

The basilar artery, in the manner in which it is formed, and in the thinness of its coats, may be considered as approximating to a vein. The similarity is increased by the peculiarity in question. Bands of the same kind are not uncommon in the longitudinal sinus, and more delicate bands and fibres are frequently met with in the heart in the right auricle, especially about the *fossa ovalis*, and in connexion with the Eustachian valve.

Whether this peculiarity of structure has a decided use, I am not prepared to say. In each instance in which a band or fibre presents itself, support is afforded, and additional strength is imparted. The band I have described, as occasionally occurring in the basilar artery, must necessarily have this effect.

It was in the month of June, last year, that my attention was first attracted to the subject. Since that time I have availed myself of every opportunity that has offered to examine the basilar artery. In ninety-eight *post-mortem* examinations at which I have been present, made in the General Hospital at Fort Pitt, during the period, I have met with it in seventeen instances. In nine, death was owing to pulmonary consumption; in two to malignant tumour; in the remainder in each instance to a different disease. The ages of the individuals varied from 19 to 59; two were 25, two 35. In point of age there was no other accordance amongst them.

I shall give the results in a tabular form, and also the proportional frequency of marked difference of size of the vertebral arteries, to which my attention was at the same time directed.

| Period. | No. of bodies examined. | Instances of band in basilar artery. | Instances of left vertebral larger than right. | Instances of right larger than left. |
|---|---|---|---|---|
| 1837. | | | | |
| June, . . . | 5 | 3 | 4 | 1 |
| July, . . . | 16 | 6 | 9 | 1 |
| August, . . | 13 | 2 | 0 | 1 |
| September, . | 9 | 1 | 2 | 3 |
| October, . . | 5 | 1 | 1 | 0 |
| November, . | 8 | 1 | 0 | 1 |
| December, . | 5 | 0 | 2 | 0 |
| 1838. | | | | |
| January, . . | 6 | 1 | 2 | 0 |
| February, . | 3 | 0 | 0 | 0 |
| March, . . | 1 | 0 | 0 | 0 |
| April, . . | 1 | 0 | 1 | 0 |
| May, . . . | 8 | 1 | 1 | 0 |
| June, . . . | 4 | 0 | 1 | 0 |
| July, . . . | 5 | 0 | 1 | 0 |
| August, . . | 9 | 1 | 2 | 1 |
| | — | — | — | — |
| | 98 | 17 | 26 | 8 |

In one instance of the total 17, a short filament proceeded from the intersecting band thus H; its end floating loosely.

In two cases out of the 98 there was another peculiarity, which I am not aware has ever been noticed, viz. an opening or foramen between the two vertebral arteries in the septum formed by their juxtaposition posterior to the basilar artery. In each instance it was sufficiently large to admit a surgeon's probe.

In three instances amongst the total number, each vertebral was as large as the basilar artery,—which was of the usual size of the artery,—and the vertebrals did not appear to be diseased.

The proportion in which the left vertebral artery was found larger than the right is so great,—viz. in the ratio of 26 to 8,—that it can hardly be considered accidental. But, on what the circumstance depends I am entirely ignorant. At one time, I supposed that it might be connected with the difference of origin of the right and left subclavian; but the notion was not supported by facts. In two instances (the only ones observed) in which the left vertebral took its origin immediately from the aorta, between the carotid and subclavian, it was smaller than the right vertebral, taking its origin from the subclavian of that side, and that subclavian, as usual, from the *arteria innominata.*—*Edin. Med. and Surg. Journ.*

*On Neuralgia of the Larynx.* By Prof. Graves.—This occurred in a young lady originally of vigorous constitution, but latterly suffering from menstrual irregularity and hysteria. The laryngeal affection had been considered to be inflammatory in the country, and had been treated with purgatives, leeches, blisters, antimonials, and finally mercurialization. No relief had been obtained, and she came to Dublin, where she was placed under my care, and that of Doctor Marsh and Mr. Barker. The pain had become almost constant when we first saw her, but was by no means violent, except now and then when it used to become suddenly aggravated. These paroxysms of pain could not even, properly speaking, be called violent; they were however, distressing, amounting to a most annoying feeling of distress about the whole region of the larynx. There was no external tenderness, and the internal fauces were healthy. We considered it to be an hysterical nervous affection. This neuralgia was chiefly remarkable for a change of tone and weakness in the voice which invariably attended the paroxysms, shewing that the *rima glottidis* and the *chordæ vocales* were the parts chiefly implicated. We must suppose, therefore, that the pain was derived from the branches of the superior laryngeal nerve, which Dr. Reid has proved to be chiefly *sensitive*.

The alteration of voice which accompanied the paroxysms of pain must be considered as a proof that the superior laryngeal nerve has some influence on the motions of the vocal organ, unless, indeed, we adopt the supposition that the affection extended likewise to the inferior laryngeal nerve. The facts of the case contain nothing decisively confirming or negativing either hypothesis.

We first gave large doses of carbonate of iron, which had the effect of rendering the attacks periodic. Every morning at *ten o'clock to the minute*, the paroxysm commenced. The dose of iron was now increased, afterwards sulphate of quinine, and finally arsenic were employed, but without any corresponding improvement. The degree of suffering became, indeed, less severe, and its duration less protracted; but it appeared extremely doubtful whether the improvement was not owing more to time than to medicine. Under these circumstances we thought it prudent to desist from all active treatment, and we recommended change of air, scenery, and the use of chalybeate mineral waters. I believe she still continues to suffer, but in a less degree. A year has now elapsed since I last saw her. This case affords a striking example of the curious fact, that medicines administered for the purpose of relieving a disease more or less fluctuating or remittent in its character, will sometimes render it strictly periodic, with marked paroxysms and free intervals. Having produced so striking an effect with our remedies, we are apt to calculate with confidence on still further improvement, and we increase the doses of tonics with boldness and full of hope; disappointment, however, here awaits us, for no tonic will be found capable of effecting any further alteration or shortening of the fit. In such cases we cannot be too much on our guard, lest we injure the constitution by too frequent attempts to procure a diminution of suffering.—*Dub. Jour. for Jan.*

---

*On Sinapisms.* By Professor Graves.—This species of rubefacient is applied in various diseases, viz. in the latter stages of fever, in pleurodynia, colic, pains of the stomach, and not unfrequently in suppressed or irregular gout, where it is attempted to fix the disease in the extremities. Nothing is more certain than that gout may go astray, and that it may, occasionally, be called away from important internal parts, by means calculated to excite inflammation on the surface. If a man, in whom a fit of the gout was about to take place, sprains his ankle, inflammation of the part is forthwith the consequence, and there the gout at once settles. Within a short period of time I have seen three remarkable examples of the relief which vital organs may experience when gout appears in the extremities. A publican applied to me with violent pain in his stomach, which came on every evening, and lasted many hours in spite of every remedy. In a day or two he got a violent attack of podagra, and had no more internal suffering. A gentleman whom I attended with Mr. Barker, was attacked with cerebral symptoms and indistinctness of vision and utterance. We feared hemiplegia; the next day he got severe podagra, and was able to speak perfectly well, and see distinctly. He was seventy-five years of age. At the very same time I was attending, with Mr. Colles and Mr. Haffield, a robust and powerfully made gentleman, aged 74 who, having had symtoms of flying gout, and shortly after a bowel complaint, made use of the salt-water plunge bath. This imprudent act brought on a violent and nearly fatal hemoptysis. He was bled twice, and got the usual styptics with relief, but his improvement became much more rapid when gout appeared in both his feet. Facts such as these occur frequently, and leave a strong impression on the mind of the practitioner of the prudence of attempting to bring the gout to the extremities in similar cases. Some try to do this by means of stuping, liniments, blisters, or sinapisms; but it appears to me that the latter are seldom applied in a manner likely to effect the desired object, for when composed of the usual ingredients, sinapisms act too quickly to be long borne, and, of course, only give rise to a very superficial inflammation, and that of very brief duration. To fix gout in a part, e. g. in the foot, our application must act much more gradually, and must excite the deeper seated tissues. These objects may be obtained by mixing one part of strong and fresh ground mustard powder with three of flour, and adding as much treacle as will convert them into a viscid paste, which may be spread like a plaster on linen, and applied to the part. This will be borne for four or six hours, and will cause a redness which will last a whole day. The proportion of flour may vary according to circumstances.—*Ibid.*

